# N-staging in large cell neuroendocrine carcinoma of the lung: diagnostic value of [^18^F]FDG PET/CT compared to the histopathology reference standard

**DOI:** 10.1186/s13550-021-00811-9

**Published:** 2021-07-22

**Authors:** Hubertus Hautzel, Yazan Alnajdawi, Wolfgang P. Fendler, Christoph Rischpler, Kaid Darwiche, Wilfried E. Eberhardt, Lale Umutlu, Dirk Theegarten, Martin Stuschke, Martin Schuler, Clemens Aigner, Ken Herrmann, Till Plönes

**Affiliations:** 1 Department of Nuclear Medicine, West German Cancer Center, University Hospital Essen, University Duisburg-Essen, Hufelandstr. 55, 45147 Essen, Germany; 2 Department of Thoracic Surgery and Endoscopy, West German Lung Center, Ruhrlandklinik - University Hospital Essen, University Duisburg-Essen, Essen, Germany; 3 Department of Pulmonary Medicine, Section of Interventional Pneumology, West German Lung Center, Ruhrlandklinik - University Hospital Essen, University Duisburg-Essen, Essen, Germany; 4 Department of Medical Oncology, West German Cancer Center, University Hospital Essen, University Duisburg-Essen, Essen, Germany; 5 Division of Thoracic Oncology, West German Lung Center, Ruhrlandklinik - University Hospital Essen, University Duisburg-Essen, Essen, Germany; 6 Department of Diagnostic and Interventional Radiology and Neuroradiology, University Hospital Essen, University Duisburg-Essen, Essen, Germany; 7 Institute of Pathology, West German Cancer Center, University Hospital Essen, University Duisburg-Essen, Essen, Germany; 8 Department of Radiotherapy, West German Cancer Center, University Hospital Essen, University Duisburg-Essen, Essen, Germany; 9 German Cancer Consortium (DKTK), Partner Site University Hospital Essen, Essen, Germany

**Keywords:** [^18^F]FDG, PET/CT, Large cell neuroendocrine carcinoma, Lung, Nodal staging

## Abstract

**Background:**

Large cell neuroendocrine carcinoma of the lung (LCNEC) is a rare entity occurring in less than 4% of all lung cancers. Due to its low differentiation and high glucose transporter 1 (GLUT1) expression, LCNEC demonstrates an increased glucose turnover. Thus, PET/CT with 2-[^18^F]-fluoro-deoxyglucose ([^18^F]FDG) is suitable for LCNEC staging. Surgery with curative intent is the treatment of choice in early stage LCNEC. Prerequisite for this is correct lymph node staging. This study aimed at evaluating the diagnostic performance of [^18^F]FDG PET/CT validated by histopathology following surgical resection or mediastinoscopy. N-staging interrater-reliability was assessed to test for robustness of the [^18^F]FDG PET/CT findings.

**Methods:**

Between 03/2014 and 12/2020, 46 patients with LCNEC were included in this single center retrospective analysis. All underwent [^18^F]FDG PET/CT for pre-operative staging and subsequently either surgery (*n* = 38) or mediastinoscopy (*n* = 8). Regarding the lymph node involvement, sensitivity, specificity, accuracy, positive predictive value (PPV) and negative predictive value (NPV) were calculated for [^18^F]FDG PET/CT using the final histopathological N-staging (pN0 to pN3) as reference.

**Results:**

Per patient 14 ± 7 (range 4–32) lymph nodes were resected and histologically processed. 31/46 patients had no LCNEC spread into the lymph nodes. In 8/46 patients, the final stage was pN1, in 5/46 pN2 and in 2/46 pN3. [^18^F]FDG PET/CT diagnosed lymph node metastasis of LCNEC with a sensitivity of 93%, a specificity of 87%, an accuracy of 89%, a PPV of 78% and a NPV of 96%. In the four false positive cases, the [^18^F]FDG uptake of the lymph nodes was 33 to 67% less in comparison with that of the respective LCNEC primary. Interrater-reliability was high with a strong level of agreement (κ = 0.82).

**Conclusions:**

In LCNEC N-staging with [^18^F]FDG PET/CT demonstrates both high sensitivity and specificity, an excellent NPV but a slightly reduced PPV. Accordingly, preoperative invasive mediastinal staging may be omitted in cases with cN0 disease by [^18^F]FDG PET/CT. In [^18^F]FDG PET/CT cN1-cN3 stages histological confirmation is warranted, particularly in case of only moderate [^18^F]FDG uptake as compared to the LCNEC primary.

## Background

Neuroendocrine tumors are rare malignancies within the lung. In particular, large cell neuroendocrine lung cancer (LCNEC) accounts for only 2–3% of all lung cancers [1, 2]. In contrast to the favorable prognosis of typical and atypical carcinoids, LCNEC presents with high biological aggressiveness similar to small cell lung cancer (SCLC) [3]. While advanced stage LCNEC is treated with systemic chemotherapy and radiotherapy, patients with early stage disease are preferentially referred to surgery followed by adjuvant chemotherapy [1, 3, 4]. Before undergoing thoracic resection with curative intent, precise staging is of utmost importance, particularly with regard to lymph node involvement and distant spread.

One characteristic of LCNEC and discriminating it from typical and atypical bronchial carcinoids is the high expression of the glucose transporter 1 (GLUT1) making this entity suitable for fluorine-18-fluoro-deoyglucose ([^18^F]FDG) imaging [5–7]. Thus, whole body staging in LCNEC is done with [^18^F]FDG PET/CT putting special emphasis on mediastinal N-staging and on distant metastases. However, since LCNEC is scarce, only few reports on the diagnostic performance of [^18^F]FDG PET/CT in this entity are published, all of them limited by small patient numbers ranging from 5 to 31 individuals [5–8]. In early stage I and II patients’ final outcome after surgery significantly depends on the lymph node status with ≥ pN1 indicating a worse prognosis [9]. Accordingly, exact non-invasive image-driven hilar and mediastinal lymph node staging by [^18^F]FDG PET/CT is of key relevance. However, up to now no data are available addressing the diagnostic accuracy of [^18^F]FDG PET/CT in this particular respect. In a monocentric setting, we retrospectively reviewed patients with LCNEC who underwent [^18^F]FDG PET/CT and subsequently either surgery with curative intent or mediastinoscopy for lymph node sampling in cases of discordant diagnostic findings. Aim of this study was to assess the performance of [^18^F]FDG PET/CT N-staging in LCNEC as compared to the lymph node histopathology representing the diagnostic gold standard.

## Methods

The local ethics review committee of the University Hospital Essen approved this retrospective analysis. The analysis was conducted in compliance with the Declaration of Helsinki.

**Fig. 1:**
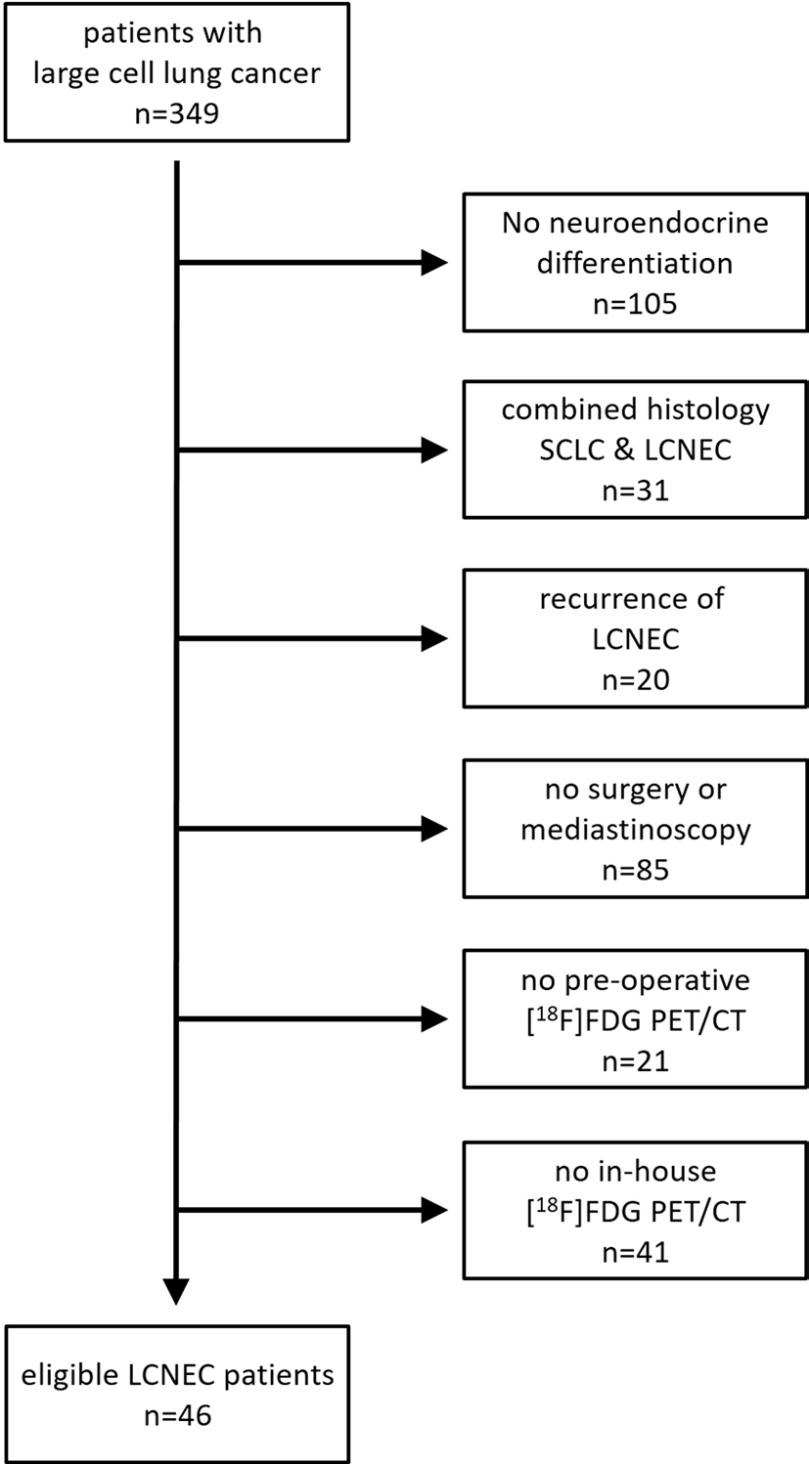
Flowchart with patient selection criteria

Between 03/2014 and 12/2020, 349 patients with large cell lung cancer were presented in our interdisciplinary tumor board (Fig. 1). Finally, 46 patients (27 female, 19 male; age 61.0 ± 8.2 years) with histologically confirmed LCNEC were identified, who received an in-house [^18^F]FDG PET/CT and subsequently underwent either surgery with curative intent (*n* = 38) or mediastinoscopy for invasive lymph node sampling (*n* = 8) due to discordant findings in PET/CT and endobronchial ultrasound-guided transbronchial needle aspiration (EBUS-TBNA). Surgery was performed as lobectomy with ipsilateral lymphadenectomy in all patients but one in whom an additional contralateral wedge resection was done to remove a solitary pulmonary metastasis in an oligometastatic setting. The removed lung lobes were entirely explored by the pathologist. That included the appearance of intrapulmonary lymph nodes (level 12–14) and their respective tumor involvement. 37/46 patients were treatment-naïve when receiving the [^18^F]FDG PET/CT. Another 9/46 had neoadjuvant chemotherapy ± radiochemotherapy and were thereafter referred for [^18^F]FDG PET/CT and subsequently for local surgical treatment in a multimodal approach.

The patients were in a fasting state at least six hours before [^18^F]FDG administration. The blood glucose level was below 160 mg/dl in all subjects. 61 ± 7 min after injection of 286 ± 51 MBq [^18^F]FDG a diagnostic PET/CT scan was acquired (*n* = 32 patients: Biograph mCT 128, Siemens Healthcare, Erlangen, Germany; *n* = 14 patients: Biograph 64 Vision 600, Siemens Healthcare, Erlangen, Germany). Both PET/CT scanners were EARL-certified, and clinical standard imaging protocols were applied. The scanned field of view comprised an area between the base of the skull and mid-thighs with the patient in a supine position. PET emission data were attenuation corrected by help of the CT data and reconstructed using time of flight technology and an iterative OSEM algorithm.

The maximum standard uptake value (SUV_max_) was measured in all primary LCNEC and in all lymph node metastases. After testing for normality (Kolmogorov–Smirnov test), the mean SUV_max_ of the primary LCNEC and the mean SUV_max_ of the histologically verified lymph node metastases were statistically compared by help of a paired *t* test (two-sided testing).

To further assess the diagnostic capabilities of [^18^F]FDG PET/CT in lymph node staging of LCNEC, all PET/CT scans were read in a blinded fashion by two experienced experts in nuclear medicine (W.F. and C.R.), both certified board members with 10 years and 9 years of experience in PET/CT reading. Lymph node staging was done visually (lymph node uptake higher than background) and by help of SUV_max_ measurements. The results were reported in consensus as N0 (no lymph node metastasis), N1 (ipsilateral peribronchial and/or hilar and/or intrapulmonary lymph node metastasis), N2 (ipsilateral mediastinal or subcarinal lymph node metastasis) or N3 (contralateral mediastinal, contralateral hilar, ipsi- or contralateral deep cervical or supraclavicular lymph node metastasis) [10, 11]. If the readers’ reports were discordant, the particular case was discussed and a final N-stage determined. Interrater-reliability was measured by calculating linear weighted Cohen’s kappa.

Finally, the PET/CT results were compared to the histopathological reference standard derived from systematic lymph node sampling or lymphadenectomy. On that base sensitivity, specificity, accuracy, positive predictive value (PPV) and negative predictive value (NPV) were calculated. False positive and false negative cases were evaluated in more detail regarding the size of the respective lymph nodes in the corresponding CT. Pathological enlargement of mediastinal and hilar lymph nodes was assessed according to the American Thoracic Society [12].

Additional subgroup analyses were performed (1) with respect to the PET/CT scanner to investigate a possible bias due to the analogue (Biograph mCT 128) versus fully digital (Biograph 64 Vision 600) technology and (2) with respect to an impact of pretreatment with chemo- ± radiotherapy on the [^18^F]FDG PET/CT results. Due to small patient numbers for these statistical subgroup comparisons, the Mann–Whitney *U* test was applied (two-sided testing).

All statistical analyses were performed with the software package Statistica version 13 (StatSoft Europe, Hamburg, Germany).

## Results

### Staging

23/46 (50%) patients had pathologic stage I disease, eleven (24%) patients stage II disease, nine (19.5%) patients stage III disease (5 × stage IIIa, 3 × stage IIIb, 1 × stage IIIc), and three (6.5%) patients were initially evaluated stage IVa disease.

### Primary tumor staging

In the T1 group (total *n* = 17, 37%), ten (22%) patients had a T1a LCNEC, five (11%) patients a T1b and two (4%) a T1c primary tumor. T2 LCNEC (total *n* = 19, 41%) divided in 15 (33%) T2a and four (8.5%) T2b tumors. Finally, seven (15%) patients had a T3 tumor and three (6.5%) patients a T4 primary.

### Lymph node involvement

With respect to the N-staging, a median of 14 ± 7 lymph nodes (range 4 to 32 lymph nodes) was removed for sampling. The overall prevalence of a lymph node involvement was 32.6%. In detail, 31 (67.5%) patients were histopathologically staged pN0, eight (17.5%) patients pN1, five (11%) patients pN2 and two (4%) pN3.

### Distant metastasis

Three patients had distant metastasis proven either by [^18^F]FDG PET/CT (contralateral pulmonary/hepatic metastasis) or by MRI (solitary brain metastasis). In an oligometastatic setting, a female patient underwent resection of the LCNEC primary and a contralateral solitary pulmonary metastasis (Fig. 3G–J). At last follow-up seven months after resection, she was free of disease. A second patient presented with a solitary brain metastasis. He received surgery for that brain metastasis and the pulmonary LCNEC primary in curative intent followed by chemo- and cerebral radiotherapy. The last patient presented with hepatic metastases and received mediastinoscopy for N-staging purposes.

### [^18^F]FDG PET findings

**Fig. 2:**
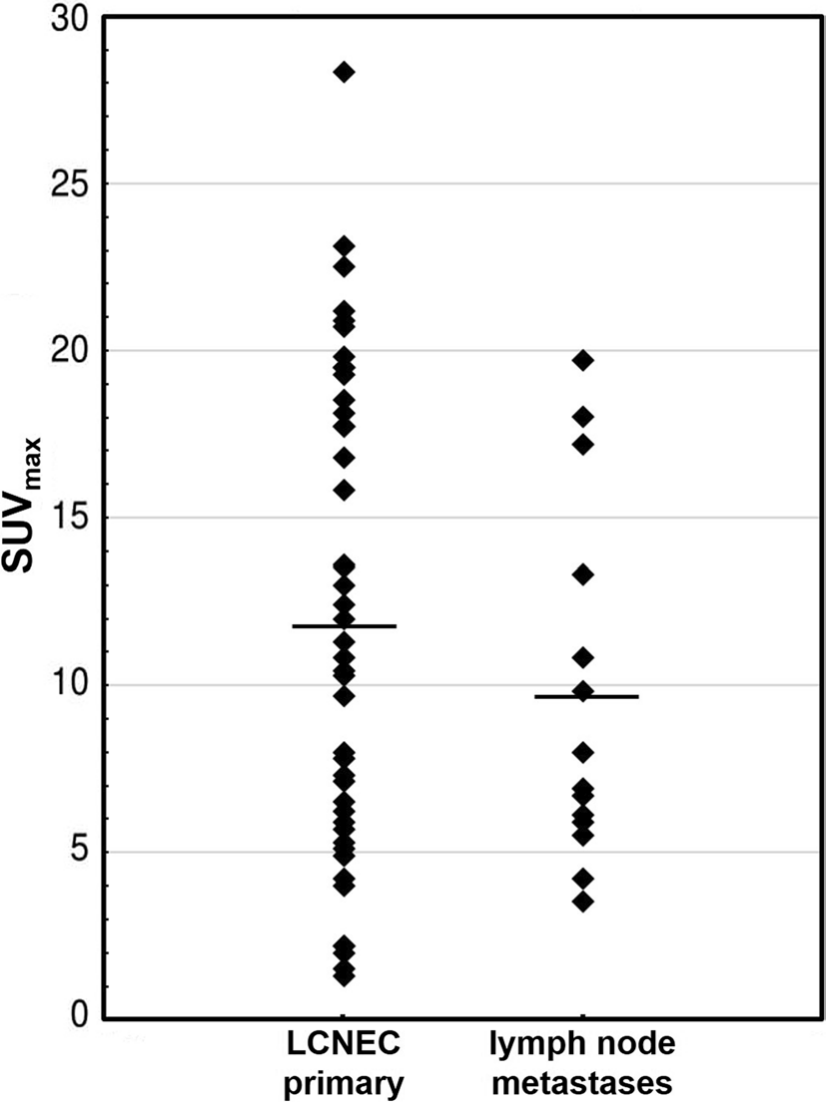
SUVmax of LCNEC primary and SUVmax of LCNEC true positive lymph nodes

[^18^F]FDG PET/CT was performed 15.7 ± 12.7 days (range 1–60 days) before surgery or mediastinoscopy. The SUV_max_ of the primary tumor was 11.9 ± 7.1 (range 1.5–28.3), while the histologically verified lymph node metastases demonstrated a SUV_max_ of 9.7 ± 5.4 (range 3.5–19.7) (Fig. 2). There was no significant difference between these uptake values (*p* = 0.29).

### [^18^F]FDG PET/CT and histopathological evaluation

When compared to the histopathological gold standard N-staging, [^18^F]FDG PET/CT was true positive in 14 patients, true negative in 27 patients, false positive in four patients and false negative in one patient (Table 1). The false negative [^18^F]FDG PET/CT missed a single infiltrated lymph node (1/5 positive lymph nodes). This patient had neoadjuvant chemotherapy, and the particular false negative lymph node demonstrated a low SUV_max_ of 2.0. However, in 2/4 of the false positive PET/CT scans, a solitary lymph node demonstrated an increased [^18^F]FDG uptake, while in the other 2/4 patients, false positive tracer turnover was found in multiple mediastinal and hilar lymph nodes.

**Table 1:** Results from histopathology and [^18^F]FDG PET/CT including sensitivity, specificity, positive and negative predictive values of [^18^F]FDG PET/CT

	Histopathology Positive	Histopathology Negative	
[^18^F]FDG PET/CT Positive	14	4	[^18^F]FDG PET/CT sensitivity: 93% [^18^F]FDG PET/CT: positive predictive value 78%
[^18^F]FDG PET/CT Negative	1	27	[^18^F]FDG PET/CT specificity: 87% [^18^F]FDG PET/CT: negative predictive value 96%

**Fig. 3:**
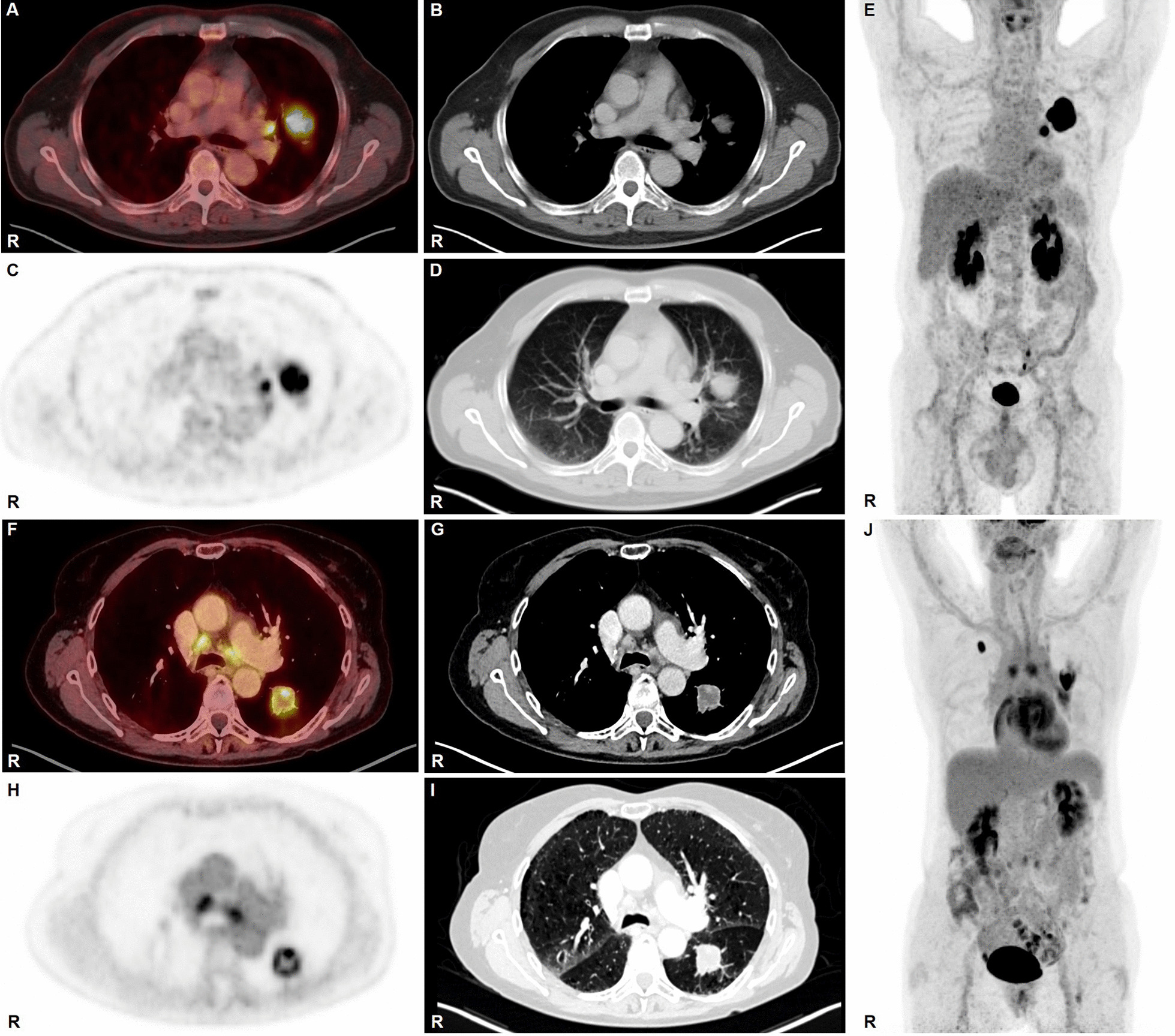
**A**–**E** true positive lymph node: [^18^F]FDG PET/CT: male, age 58 years, LCNEC pT3 pN1 (1/11) M0, stage IIIa. SUV_max_ primary 17.7, SUV_max_ true positive lymph node metastasis 10.8. **A** [^18^F]FDG PET/CT fusion, **B** CT chest soft tissue window, **C** PET attenuation-corrected emission, **D** CT chest lung window, **E** PET maximum intensity projection (MIP). **F**–**J** false positive lymph node: [^18^F]FDG PET/CT: female, age 66 years, LCNEC pT1c pN0 (0/21) pM1a (resected single metastasis in contralateral lung), stage IVa. Patient underwent EBUS-TBNA 3 days prior to FDG PET/CT. SUV_max_ primary 15.8, SUV_max_ false positive lymph nodes 5.2. **F** [^18^F]FDG PET/CT fusion, **G** CT chest soft tissue window, **H** PET attenuation-corrected emission, **I** CT chest lung window, **J** PET maximum intensity projection (MIP). Last follow-up 7 months after initial diagnosis: no evidence of recurrence

Taken together, this translates into a sensitivity of 93%, a specificity of 87%, an accuracy of 89%, a positive predictive value of 78% and a negative predictive value of 96% (Table 1). Figure 3 depicts examples for a true positive and a false positive [^18^F]FDG PET/CT scan, respectively (Fig. 3).

The addition of the CT-derived lymph node size to the PET/CT decision of a nodal involvement neither contributed to a better sensitivity nor specificity. In detail, in the false negative patient, the positive lymph node was located at 4L and was CT-morphologically not enlarged with a short axis of 7 mm. Contrary, in three of the four false positive patients, the respective lymph node was also enlarged by CT-morphological means (case 1: lymph node at 11L, short axis 13 mm; case 2: lymph node at 4L, short axis 12 mm; case3: lymph node at 4R, 11 mm). The fourth patient was staged cN3 by [^18^F]FDG PET/CT but in fact was pN1 after mediastinoscopy and subsequent surgery. Taking the additional CT information into account, the mediastinal lymph nodes were still rated positive with a short axis of 12 mm at location L7 but negative at the contralateral hilar region 11R with a short axis of 9 mm. That translated in a clinical down staging by the CT-component of the PET/CT from N3 to N2. However, true pathological stage was N1, and this particular case remained false positive at the per-patient level.

### Interrater variability

Analysis of the interrater-reliability regarding the N-staging revealed a strong level of agreement with κ = 0.82 between both readers (*p* < 0.001) [13]. In eight patients, the lymph node ratings of the readers were discordant.

### PET/CT scanner subgroup analysis

32 patients were investigated using the PET/CT scanner with analogue technique and 14 patients underwent the [^18^F]FDG scan in the fully digital PET/CT machine. Analyses of the [^18^F]FDG uptake in LCNEC primary tumors demonstrated a SUV_max_ of 12.1 ± 7.9 in the analogue PET/CT group and 11.3 ± 4.9 in the digital PET/CT group. For the true positive lymph node metastases SUV_max_ findings were 10.1 ± 5.5 in the analogue PET/CT group and 9.1 ± 5.6 in the digital PET/CT group, respectively.

Mann–Whitney U-statistics revealed no significant differences between the two scanner types.

Calculations of sensitivity, specificity and accuracy resulted in 88%, 88% and 88%, respectively, in the analogue PET/CT group and in 100%, 88% and 93%, respectively, in the digital PET/CT group.

### Pretreatment subgroup analysis

In total, 37 patients directly underwent surgery as first therapy, while nine patients received preoperative treatment with chemotherapy ± radiotherapy. In the surgery group, the SUV_max_ of the LCNEC primary tumor was 11.8 ± 6.5 and that of the true positive lymph node metastases 10.3 ± 5.6. In the group with neoadjuvant treatment, the SUV_max_ of the primary tumor was 12.4 ± 9.7. In those two patients with pretreated true positive lymph node metastases, SUV_max_ was 6.7 and 5.5, respectively. Mann–Whitney U-statistics revealed no significant differences between these subgroups.

Calculation of sensitivity, specificity and accuracy resulted in 100%, 85% and 89%, respectively, in the surgery first group and in 67%, 100% and 89%, respectively, in the chemo- ± radiotherapy pretreatment group.

## Discussion

To the best of our knowledge, this is the first investigation focusing on [^18^F]FDG PET/CT and N-staging of the rare entity LCNEC. Our study revealed high sensitivity and specificity of [^18^F]FDG PET/CT in lymph node staging of LCNEC patients as compared to the histopathological gold standard. This excellent performance is of fundamental relevance as LCNEC patients in early stages (I to III) benefit from surgery with curative intent [4]. In contrast to the less malignant typical and atypical bronchial carcinoids, LCNEC demonstrates a substantially higher proliferation rate accompanied by a generally increased glucose consumption making this entity an important target for [^18^F]FDG PET/CT imaging [8, 14]. In a cohort of 61 LCNEC, Grøndahl et al. reported 100% [^18^F]FDG avidity for the primary [15]. In addition, SUV_max_ of the primary LCNEC is a predictive factor for overall survival [6, 8]. Our SUV_max_ of 11.9 ± 7.1 in LCNEC primaries is in good agreement with all previous studies [5, 6, 8, 16]. This provides further evidence that [^18^F]FDG PET/CT plays a key role in staging of LCNEC. In addition, the mean SUV_max_ of LCNEC primaries appears to be higher than those of adenocarcinomas (e.g., Agarwal et al. SUVmax 4.5; Casali et al.: SUVmax 5.2 ± 0.3) but comparable to that of squamous cell lung cancer (e.g., Agarwal et al. 2010: SUV_max_ 9.5; Casali et al. 2010 SUV_max_ 10.5 ± 0.9) [16, 17].

The intense [^18^F]FDG turnover in LCNEC primaries indicates that [^18^F]FDG PET/CT is also suitable for N-staging with rather robust findings. First of all, this is supported by fact that the SUV_max_ of the LCNEC primary and the corresponding lymph node metastases are not significantly different. Secondly, with respect to N-staging our analyses revealed the following: sensitivity 93%, specificity 87%, PPV 78%, NPV 96% and accuracy 89%. This underlines that [^18^F]FDG PET/CT is a reliable diagnostic tool for LCNEC N staging. In addition, the interrater-reliability is strong with respect to the diagnosis and extent of a lymph node involvement.

Going into more detail, regarding the false N-staging results, it became evident that in the false negative case the primary LCNEC had a quite low glucose consumption. This patient underwent neoadjuvant chemotherapy which apparently reduced the glucose metabolism significantly. Therefore, the a priori sensitivity for detecting lymph node or distant tumor spread in that particular patient was rather reduced and, consequently, the [^18^F]FDG PET/CT missed the lymph node metastasis. The four false positive cases were treatment naïve prior to the [^18^F]FDG PET/CT. In all these patients, the SUV_max_ of the lymph nodes which were deemed suspicious for metastases was 33 to 67% less in comparison with that SUV_max_ of the respective LCNEC primary. This is in contrast to the results of the true positive lymph node metastases which demonstrated a mean SUV_max_ not statistically lower than that of the corresponding primary tumor. Taken together, the interpretation of a lymph node as metastasis should be put forward with caution whenever its SUV_max_ is considerably lower than that of its LCNEC primary. In addition, increases in [^18^F]FDG uptake might be induced by inflammatory changes in those lymph nodes either due to additional lung comorbidities, peritumoral changes or artificially due to bronchoscopic procedures a few days prior to the PET/CT [18]. The latter was the case in 2/4 false positives who underwent EBUS TBNA two and three days prior to the [^18^F]FDG PET/CT. A third patient was rated N3 by FDG PET/CT but in fact was histopathologically N1. Next to one ipsilateral hilar lymph node metastasis, the mediastinoscopy revealed benign lymph nodes with histiocytic reaction. This feature is known to increase the [^18^F]FDG uptake into mediastinal and hilar lymph nodes via GLUT1 overexpression leading to false positive results [19]. In the last patient rated false positive, the primary LCNEC demonstrated only a moderately increased glucose consumption with a SUV_max_ of 4.0. In an adjacent ipsilateral hilar lymph node, the SUV_max_ was measured 2.7 which was interpreted as N1 stage. However, mediastinoscopy and subsequent surgery revealed no lymph node involvement (0/15). In addition, the CT-morphologically derived lymph node size as an additional criterion for more accurate identification of lymph node involvement could not overcome the shortcomings of the metabolic PET staging in our cohort.

Contrary to the high sensitivity and specificity of [^18^F]FDG PET/CT N-staging in LCNEC, in non-small cell lung cancers (NSCLC) Gedik et al. questioned the accuracy of [^18^F]FDG PET/CT for N-staging [20]. They reported an overall accuracy of only 62% as compared to surgical N-staging and attributed their high rate of false positive findings to inflammatory processes. In addition, the high rate of false negative N-stagings was seen in the light of close neighboring of NSCLC primary and hilar lymph node metastasis as well as in insufficient spatial resolution of the PET/CT scanner used in their study. Likewise, Bustos García de Castro et al. investigated the performance of [^18^F]FDG PET/CT N-staging in NSCLC in comparison with histopathology and demonstrated limited sensitivity of 54% and specificity of 77% [21]. Additional data from Darling et al. also point to a considerably impaired performance of [^18^F]FDG PET/CT in N-staging of NSCLC, especially in terms of sensitivity (70%), while the specificity (94%) was even higher when compared to our findings in LCNEC [22]. One reason for the higher sensitivity of [^18^F]FDG PET/CT in N-staging of LCNEC might be the high GLUT1 expression paralleled by an increased glucose consumption in metastatic LCNEC lymph nodes as compared to metastatic NSCLC lymph nodes [7]. In this respect, Lee et al. demonstrated only moderate [^18^F]FDG uptake especially in lymph node metastases of adenocarcinomas which was significantly less than that of lymph node metastases from squamous cell carcinomas [23]. However, Xue et al. reported more favorable results for N-staging in 112 NSCLC patients undergoing [^18^F]FDG PET/CT and subsequent surgery [24]. They found a sensitivity of 92% and a specificity of 93%, which are comparable to our LCNEC data. Anatomically detailed analyses of Zhang et al. in 83 NSCLC patients depicted a sensitivity of 80% and a specificity of 86% for lymph nodes at locations 2R, 2L, 4R, 4L and 7 but sensitivity dropped to 42% for lymph nodes at stations 3, 5, 6, 8, 9 and 10, while specificity remained at 88% [25]. Furthermore, they found that [^18^F]FDG PET/CT sensitivity for N-staging was excellent in small T0 to T2 primaries (100%) but was impaired in larger T3 and T4 primaries. Due to the overall high negative predictive values of 86 to 100% in their series, they concluded that in NSCLC patients a negative mediastinal N-staging in terms of PET/CT might prevent patients from additional invasive mediastinoscopy. With respect to the NPV of 96% in our study group, one might suggest the same for patients with LCNEC and negative mediastinal [^18^F]FDG PET/CT N-staging.

Despite the rather robust results for [^18^F]FDG PET/CT in LCNEC, more tumor-specific radiotracers are searched for in the context of lung cancer to especially overcome false positive results due to benign inflammatory processes. ^18^F-fluorothymidine ([^18^F]FLT) directly targets the cell proliferation of NSCLC, while the tumor-related angiogenesis can be visualized by radiolabeled integrin αvβ3 antagonists via arginine-glycine-aspartic acid (RGD) peptides [26]. One future candidate for an even more precise N- and M-staging is the chemokine receptor CXCR4 and its radiolabeled ligand [^68^Ga]pentixafor which addresses tumor growth, invasiveness and metastasis [27]. Another approach is tumor-associated fibroblasts which can be labeled via the fibroblast activation protein (FAP) with [^68^Ga]FAPI inhibitors [28].

There are limitations of our study which need to be addressed: firstly, its retrospective and monocentric design. In addition, early stage LCNEC referred to either mediastinoscopy or surgery under curative intent is quite rare. This lowers the number of patients to be included in a comparative [^18^F]FDG PET/CT vs. histopathology study. Furthermore, neoadjuvant treatment in a subset of patients before [^18^F]FDG PET/CT and subsequent surgical N-staging might contribute to within-group heterogeneity. Though, a subgroup comparison between patients with versus without neoadjuvant treatment revealed neither significant differences in [^18^F]FDG accumulation nor in [^18^F]FDG PET/CT accuracy. Solely the sensitivity in detecting lymph node metastases might drop in pretreated patients. Finally, the different PET/CT scanners might have affected the results as technology evolves. Thus, the impact of PET/CT scanner generation was also tested in a further subgroup analysis. As expected, since both scanners were EARL-certified, this analogue versus digital PET/CT comparison also revealed no significant differences in the SUV_max_ of LCNEC primaries and lymph node metastases, respectively, and resulting sensitivities and specificities were comparable. However, larger preferably multicentric studies with state-of-the-art PET/CT scanners are mandatory to further explore the non-invasive staging abilities of [^18^F]FDG PET/CT in the context of LCNEC. As [^18^F]FDG PET/CT will continue to be the diagnostic mainstay in LCNEC staging for the next years, a future prospective study might have high impact on the new guidelines and patient care. Therefore, inclusion and exclusion criteria ought to be strict. In particular, EBUS-TBNA prior to PET/CT needs to be avoided. Furthermore, in a first step only therapy-naïve patients should be included and those patients with known inflammatory diseases involving intrathoracic lymph nodes like sarcoidosis, silicosis or anthracosis should be excluded. Finally, a standardized read of the [^18^F]FDG PET/CTs with adherence to predefined anatomical lymph node levels will contribute to clarifyication of the role of [^18^F]FDG PET/CT in LCNEC staging.

## Conclusions

[^18^F]FDG PET/CT is a reliable tool for N-staging of LCNEC with high sensitivity, specificity and NPV. In case of a negative [^18^F]FDG PET/CT in terms of N-stage additional invasive staging procedures might be omitted, especially in therapy naïve patients. However, lymph nodes with only moderately increased glucose consumption are candidates for invasive staging to establish a final histological verification as other pathologies like inflammation or shortly preceding diagnostic interventions may trigger this tracer uptake.

## Data Availability

The datasets generated and analyzed during the current study are not publicly available due to privacy legislation but are available from the corresponding author on reasonable request.

## Abbreviations

LCNEC: Large cell neuroendocrine lung cancer
NSCLC: Non-small cell lung cancer
SCLC: Small cell lung cancer
[^18^F]FDG: [^18^F]-fluoro-deoxyglucose
PET: Positron emission tomography
CT: Computer tomography
PPV: Positive predictive value
NPV: Negative predictive value
GLUT1: Glucose transporter 1
SUV_max_: Maximum standard uptake value
EBUS-TBNA: Endobronchial ultrasound-guided transbronchial needle aspiration
MRI: Magnetic resonance imaging
FLT: Fluorothymidine
CXCR4: Chemokine receptor
FAP: Fibroblast activation protein
